# Is there a rationale for perioperative nutrition therapy in the times of ERAS?

**DOI:** 10.1515/iss-2019-0012

**Published:** 2019-11-30

**Authors:** Arved Weimann

**Affiliations:** Klinik für Allgemein-, Viszeral- und Onkologische Chirurgie mit Abteilung Klinische Ernährung, Klinikum St. Georg gGmbH Leipzig, Delitzscher Str. 141, 04129 Leipzig, Germany

**Keywords:** cancer patient, conditioning, Enhanced Recovery after Surgery (ERAS), neoadjuvant treatment, nutrition therapy, perioperative, prehabilitation

## Abstract

In order to increase patient compliance in Enhanced Recovery after Surgery (ERAS) programs, assessment and monitoring of functional and nutritional status should be routinely performed. Sarcopenic obesity is frequently underestimated and has been shown to be a significant risk factor for the development of postoperative complications. With special regard to gastrointestinal cancer patients undergoing neoadjuvant treatment, nutritional deficiencies may develop stepwise and increase during therapy. In the case of proven deficits, recent strategies including “prehabilitation” focus on making the patient fit for an ERAS program. Evidence-based guidelines for perioperative nutrition therapy have been available.

## Zusammenfassung

Auch in Zeiten von ERAS wird die präoperative Erfassung von Ernährungsstatus und Funktionalität mit dem Ziel empfohlen, die Patientencompliance im Programm zu verbessern. Gerade die sarkopene Adipositas wird häufig unterschätzt, hat sich aber als signifikanter Risikofaktor für das Entstehen postoperativer Komplikationen erwiesen. Beim gastrointestinalen Karzinompatienten können sich im Rahmen einer multimodalen Therapie Defizite weiter verstärken, bei deren Vorliegen eine präoperative „Prähabilitation“ zu erwägen ist. Unter Einschluss einer Ernährungsintervention zielt die multimodale Prähabilitation auf die Konditionierung für ein ERAS Programm. Evidenz basierte Leitlinien für die perioperative Ernährungstherapie dieser Patienten liegen vor.

## Introduction

In the time of Enhanced Recovery after Surgery programs (ERAS), perioperative nutrition therapy seems to be very “traditional” and even redundant. However, ERAS also focuses on metabolism including in the bundle of treatment modalities nutrition as well. It is also appropriate for patients at risk like the elderly [1], [2], [3]. A nutritional goal is the prevention of deterioration of the nutritional status. From a metabolic and nutritional point of view, the European Society for Clinical Nutrition in Surgery (ESPEN) guidelines are in line with ERAS and include the following [4]:

– integration of nutrition into the overall management of the patient
– avoidance of long periods of preoperative fasting
– re-establishment of oral feeding as early as possible after surgery
– start of nutritional therapy early, as soon as a nutritional risk becomes apparent
– metabolic control e.g. of blood glucose
– reduction of factors which exacerbate stress-related catabolism or impair gastrointestinal function
– minimized time on paralytic agents for ventilator management in the postoperative period
– early mobilization to facilitate protein synthesis and muscle function

The recent ERAS guideline update also recommends correction of derangements such as anemia and malnutrition [5]. High compliance to ERAS protocols may be associated with improved 5-year cancer-specific survival after major colorectal surgery [6]. However, the implementation is not easy and may be optimized as had been demonstrated in a recent study from several European countries [7].

Recent observational data show that patients with high ERAS compliance consumed more protein. Consumption of ≥60% protein needed after surgery and Malnutrition Screening Tool scores were independent predictors of length of hospital stay [8].

In a recent ERAS implementation study from Canada, nutritional risk measured by the Nutritional Risk Score (NRS) predicted low overall compliance (<70%) to ERAS (OR 2.77; 95% CI 2.11–3.64); p<0.001 and a trend for LOS >5 days (OR 1.40; 95% CI 1.00–1.96); p=0.052. Low compliance to ERAS (<70%) predicted postoperative complications (OR 2.69; 95% CI 2.23–3.24); p<0.001 [9].

## Surgical risk and functional status

With special regard to the elderly functional status and dependency, cognitive function and nutritional status have been proven to be significantly associated with postoperative morbidity and mortality [10], [11]. Preoperative geriatric assessment before surgery including cognitive and nutritional status should be the new standard of care. Appropriate and validated tools and scores are available [12].

## Sarcopenic obesity

Next to age-related comorbidity, functional status is determined by muscle mass which is a key component of nutritional status. From a metabolic point of view, a special risk combination for postoperative complications is the impairment of functional and nutritional status. This is a classical issue in patients undergoing surgery, and the risk to neglect had been emphasized by the pioneer in surgical metabolism Graham L. Hill many years ago [13].

Nowadays, most surgical patients are overweight or obese. Even in overweight and obese patients impaired body composition may occur. Deficiency in muscle mass and strength – so called sarcopenia – has been considered to have considerable impact on postoperative function regarding mobilization and respiratory parameters.

A meta-analysis from 28 studies showed a significantly increased risk in sarcopenic patients for major (p<0.0001) and total postoperative complications (p=0.001) [14]. In 750 obese patients with signs of malnutrition, an in-hospital mortality of 7% was found [15]. In surgical cancer, patient’s nutritional deficiencies are more frequently related to sarcopenia than to low body mass index associated with advanced tumor stage, e.g. in the upper gastrointestinal cancer.

## New definition of malnutrition

In 2018, the Global Leadership Initiative on Malnutrition has presented a new consensus for the definition of malnutrition endorsed by the major clinical nutrition societies worldwide [16]. As a two-step approach, phenotypic and etiologic criteria have to be fulfilled.

Phenotypic criteria:

– Non volitional weight loss
– Low body mass index
– Reduced muscle mass

Etiologic criteria

– Reduced food intake or assimilation
– Inflammation
– Disease burden

In line with these criteria and according to the ESPEN guidelines, severe metabolic risk can be defined in case of at least one of the following criteria [4].

– Weight loss >10–15% within 6 months
– Body mass index <18.5 kg/m^2^
– Subjective Global Assessment Grade C or NRS ≥5
– Preoperative serum albumin <30 g/L (with no evidence of hepatic or renal dysfunction).

Preoperative serum albumin is a prognostic factor for complications after surgery [17] and also associated with impaired nutritional status. Therefore, albumin may also be considered to define surgical patients at metabolic risk.

A persistently low, even decreasing or increasing serum albumin concentration is a good parameter of whether recovery is successful or not [18]. The magnitude of the postoperative systemic inflammatory response shown in the C-reactive protein may be even significantly associated with long-term outcome after colorectal surgery independent of postoperative complications or disease stage [18].

## Routine computerized tomography for the assessment of body composition

Quantitative analysis of muscle in abdominal cross sections on the level of L3 has shown good correlation with muscle and fat mass of the whole body. During the past years numerous studies have clearly shown the prognostic impact of computerized tomography (CT)-derived body composition in oncological patients and those undergoing cancer surgery. L3 skeletal muscle index can be considered to be a surrogate marker for sarcopenia [19], [20]. For the measurement, two software tools are available: ImageJ des National Institute of Health (Bethesda, MD, USA) and Siliceomatic (TomoVision), Montreal, Quebec, Canada. Because sarcopenia and myosteatosis are prevalent in overweight and obese cancer patients, it is reasonable to assess muscle mass using CT [21]. With special regard to cancer patients undergoing neoadjuvant treatment, CT scans are usually performed several times during the course of treatment. It may be reasonable to implement this tool for additional assessment of body composition in clinical practice.

## Indication for nutrition therapy

The ESPEN guideline [4] state as a good clinical practice recommendation: “Perioperative nutritional support therapy is indicatedin patients with malnutrition and those at nutritional risk. Perioperative nutritional therapy should also be initiated, if it is anticipated that the patient will be unable to eat for more than 5 days perioperatively. It is also indicated in patients expected to have low oral intake and who cannot maintain above 50% of recommended intake for more than 7 days. In these situations, it is recommended to initiate nutritional support therapy (preferably by the enteral route) without delay.” Following guideline recommendations, perioperative nutrition therapy should be also part of an ERAS program and focus on avoiding perioperative weight loss.

## Prehabilitation

In case of obvious functional and nutritional deficits, “prehabilitation” offers a new concept of conditioning making the patient fit for ERAS [22]. Prehabilitation is especially appropriate in the time interval after neoadjuvant treatment before surgery.

After neoadjuvant therapy the time period for recovery before surgery is about 4–6 weeks. So far, structured preparation for surgery for several weeks is uncommon. This period may be used for conditioning the patient in a prehabilitation program with endurance and resistance exercise training. Prehabilitation modules are also nutritional and psychological therapy [22]. First results showed significant improvement of cardiopulmonary parameters with diminished oxygen consumption and improvement of quality of life. Regarding postoperative complications and outcome in colorectal cancer patients and those undergoing liver resection, no significant benefit could be found [23], [24]. However, in colorectal cancer patients prehabilitation significantly reduced the surgical stress-induced loss of lean body mass when compared with rehabilitation interventions starting after surgery [25]. A randomized blinded controlled trial investigated personalized prehabilitation in 125 high-risk patients undergoing elective major abdominal surgery. Inclusion criteria were age >70 years and/or ASA (American Society of Anesthesiologists) score III/IV. The results are promising. Patients suffering from postoperative complications, number of complications per patient, and medical complications were significantly lower in the prehabilitation group (p=0.001) [26].

The major interest on this topic of high clinical importance is clearly illustrated by seven very recent systematic reviews and meta-analyses [22], [27], [28], [29], [30], [31], [32]. Summarizing these results there is significant heterogeneity between studies. Suitable target populations and optimum protocols including appropriate supervision have to be defined. Long-term results are missing. Most likely, high-risk patients with functional and nutritional impairment will benefit most. It remains to be elucidated whether other modules should be added whenever appropriate. There is growing evidence that prehabilitation may decrease complications and shorten hospital length of stay. Results from ongoing trials have to be awaited. In Germany, appropriate outpatient modalities in the framework of interprofessional cooperation reimbursed by the health care insurances are pending.

## Oral nutritional supplements (ONS)

It is a key intervention of ERAS that preoperative fasting should be minimized and postoperatively patients should be encouraged to take normal food as soon as possible after surgery. ONS can be used for supplementing total intake and have shown in a recent meta-analysis significant impact on the decrease of postoperative complications and the hospital length of stay [33]. More data from controlled trials are needed with special regard to the postoperative patient after discharge.

Oral nutritional supplements may be also used for prehabilitation. The ESPEN guidelines state as an A recommendation “Preoperatively, oral nutritional supplements shall be given to all malnourished cancer and high-risk patients undergoing major abdominal surgery. A special risk group are the elderly people with sarcopenia” (A) [4].

## Metabolic conditioning

Metabolic conditioning – the so called “carbohydrate loading” – means a glucose drink focusing on the perioperative normoglycemia with special regard to the avoidance of postoperative insulin resistance and the reduction of perioperative discomfort. In the guidelines carbohydrate loading is recommended on the night before (200 mL) and 2 h before surgery (100 mL). A recent meta-analysis including 43 trials with 3110 patients showed a small reduction of hospital length of stay in comparison with fasting only. No benefit was observed in comparison with water and placebo. No reduction in postoperative complication rate was found [34]. It has to be argued that a considerable number of studies had included patients with minor surgery and very short hospital length of stay. The most recent multicenter randomized study from Italy included 662 patients. While significantly less patients had the requirement of 1 dose insulin/day and blood glucose levels >140 mg/dL, no difference in clinical complications could be found [35].

The ESPEN guideline recommendation focuses on patients undergoing major surgery. “In order to reduce perioperative discomfort including anxiety oral preoperative carbohydrate treatment (instead of overnight fasting), the night before and 2 h before surgery should beadministered (B)(QL). To impact postoperative insulin resistance and hospital length of staymetabolic conditioning can be considered in patients undergoing major surgery (0)” [4].

## Immunologic conditioning

The stimulation of immune defense by appropriate nutrition – called “immunonutrition” – is a challenging concept with special regard to the conditioning of patients undergoing major cancer surgery. Stimulation of T-cell antitumoral activity has been shown by arginine [36], [37]. For the combination of arginine, omega-3-fatty acids, and ribonucleotides numerous prospective randomized controlled studies and meta-analyses have been performed investigating the pre-, peri-, and postoperative use. Significant advantages regarding the cost-benefit analysis had been shown as well [38].

The sole administration before surgery revealed significant benefits in comparison with a regular hospital diet in a former meta-analysis. However, no advantage was found for the comparison with a standard oral nutritional supplement [39]. A very recent meta-analysis focused on patients undergoing gastrointestinal cancer surgery and included data from 16 randomized controlled trials with 1387 (n=715 immunonutrition and n=672 control group). In this meta-analysis the sole use of immunonutrition before surgery again led to a significant decrease of infectious complications when compared with normal diet but also with isonitrogenous standard nutritional supplement (OR 0.52; 95% CI 0.38–0.71, p<0.0001). For the hospital length of stay a significant reduction was found for immunonutrition vs. hospital diet, and a tendency vs. standard nutritional supplement [40].

The ESPEN guideline recommends the intake of oral nutritional supplements before major surgery, while immunomodulating substrates should be preferred for 5–7 days. Aiming to decrease postoperative infection rate, the available data also emphasize continuation of immunonutrition after surgery for 5–7 days [4].

Immunonutrition was also investigated within an ERAS program. In a randomized controlled study in 264 patients undergoing colorectal surgery, a diet enriched with immunonutrients was compared with a standard oral nutritional supplement administered 7 days before surgery and continued for 5 days postoperatively. In the immunonutrition group a significant decrease in the rate of infectious complications was found (23.8% vs. 10.7%; p=0.0007) [41]. Therefore, the integration of immunonutrition in an ERAS protocol may be considered.

## Conclusion

In order to increase patient compliance in ERAS, functional and nutritional status should not be ignored and needs critical observation. With special regard to gastrointestinal cancer patients, sarcopenic obesity may be underestimated. In case of proven deficits, a “prehabilitation” program has to be considered. Recent guidelines for preoperative nutrition therapy have been available and focus on making the patient fit for ERAS.

## Supplementary Material

The article (https://doi.org/10.1515/iss-2019-0012) offers reviewer assessments as supplementary material.
