# Supplementary material for: Is there a rationale for perioperative nutrition therapy in the times of ERAS?
Source: Innov Surg Sci. 2019 Nov 30;4(4):152–7. doi: 10.1515/iss-2019-0012 (PMC8059352; doi:10.1515/iss-2019-0012)
Supplement: Supplementary file 1 [file iss-04-20190012-s001.pdf]

Reviewer Assessment

Arved Weimann*

Is there a rationale for perioperative nutrition therapy in the times of ERAS?

https://doi.org/10.1515/iss-2019-0012
Received July 14, 2019; accepted September 16, 2019

***Corresponding author: Prof. Dr. Arved Weimann,** MA, Klinik für Allgemein-, Viszeral- und Onkologische Chirurgie mit Abteilung Klinische Ernährung, Klinikum St. Georg gGmbH Leipzig, Delitzscher Str. 141, 04129 Leipzig, Germany, Phone: +49/341 909-2200, E-mail: Arved.Weimann@sanktgeorg.de. https://orcid.org/0000-0001-5474-2171

Editor Comments to Original Submission

**Recommendation Term:**	**Accept**
**Overall Reviewer Manuscript Rating:**	**80**

This is a concise overview of the current knowledge on perioperative Nutrition in visceral surgery and should be published in its current state.

Open Access. © 2019 Weimann A., published by De Gruyter. This work is licensed under the Creative Commons Attribution 4.0 Public License.
